# The role of urine neutrophil gelatinase – associated lipocalin (NGAL) in acute heart failure in patients with ST – elevation myocardial infarction

**DOI:** 10.1186/s12872-015-0054-9

**Published:** 2015-06-13

**Authors:** Simona Kirbiš, Maksimiljan Gorenjak, Andreja Sinkovič

**Affiliations:** Department of intensive care unit, University Clinical Center Maribor, Ljubljanska 5, Maribor, SI 2000 Slovenia; Department of laboratory diagnostics, University Clinical Center Maribor, Maribor, Slovenia

**Keywords:** Neutrophil gelatinase-associated lipocalin - NGAL, Heart failure, Myocardial infarction, Acute kidney injury

## Abstract

**Background:**

Neutrophil gelatinase-associated lipocalin (NGAL) is a novel early marker of acute kidney injury for which has been shown that it can also be released from the injured myocardium. Our aim was to correlate urine NGAL with markers of in-hospital heart failure in patients with acute ST-elevation myocardial infarction (STEMI).

**Methods:**

We prospectively included 61 consecutive STEMI patients after primary percutaneous coronary intervention and estimated admission and in-hospital urine NGAL, serum creatinine, troponin I, leucocytes, CRP, N-terminal pro brain natriuretic peptide (NT-proBNP) levels and ejection fraction by echocardiography. Urine NGAL levels were compared between patients with and without HF defined as serum NT-proBNP > 400 pmol/l and were correlated to markers of heart failure, inflammations and of kidney function.

**Results:**

Urine NGAL levels and CRP was significantly higher in participants with heart failure compared to those with NT-proBNP below 400 pmol/l. Urine NGAL level of 50 ng/ml had 90 % specificity for HF, the sensitivity was low at 25 %. Comparison of participants with NGAL levels < 50 ng/ml and ≥ 50 ng/ml at admission and after 12 h revealed a significant difference in NT-proBNP levels, left ventricle ejection fraction, markers of inflammation and of kidney function. Urine NGAL level was independently associated with NT-proBNP level.

**Conclusions:**

The level of urine NGAL early after myocardial infarction is associated with NT-proBNP concentration and even NGAL levels below 137 ng/ml, the usually reported normal cut-off value, had high specificity for HF in our sample.

## Background

In spite of early contemporary reperfusion therapy by primary percutaneous coronary intervention (PPCI), ST-elevation myocardial infarction (STEMI) can lead to systolic and/or diastolic dysfunction with decreased cardiac output, leading to further decrease in systemic and coronary perfusion and activation of inflammation. It has been shown that inflammation plays an important role in acute heart failure (HF) after acute myocardial infarction (MI) and there is evidence for profound neutrophil activation during acute MI with release of proinflammatory markers [1, 2]. Inflammation is also important in the process of remodeling by influencing matrix degeneration and fibrosis, promoting apoptosis etc. [3–5].

Neutrophil gelatinase-associated lipocalin (NGAL) is a 25 kDa glycoprotein of the lipocalin superfamily, synthesized in granulocyte precursors in bone marrow during a narrow window of their maturation. It is stored in specific granules of mature neutrophils in complex with gelatinase [6, 7]. In recent years, NGAL has been considered mainly a predictor of acute kidney injury (AKI), because its plasma and urine levels rise before any increase in creatinine level is encountered [8]. In addition, NGAL has been associated with cell death, inflammation and matrix degradation and there is increasing evidence for enhanced systemic and myocardial expression of NGAL after acute MI, supporting the role of inflammation in this entity [9–12].

The aim of our present study was to evaluate the association of urine NGAL early after acute STEMI with markers of myocardial dysfunction and test its predictive value for acute HF.

## Methods

### Study design and participants

We performed an observational prospective monocentric study with patients admitted with acute STEMI to the Department of Medical Intensive Care (ICU), University Medical Centre Maribor. The study was approved by the Republic of Slovenia National Medical Ethics committee (77/01/11) and written informed consent was obtained from all patients. The study protocol conformed to the ethical guidelines of the Declaration of Helsinki.

Consecutive patients admitted with STEMI between April 2010 and July 2011 who had PPCI performed and stayed at the ICU for at least 24 h were included in the study. Criteria for STEMI were ischemic chest pain lasting up to 12 h plus persistent ST-elevation or new bundle-branch block on ECG [13]; PPCI is the primary reperfusion strategy for acute STEMI at our institution. Exclusion criteria were prior malignancy, infectious disease, sepsis and death or discharge from the ICU in less than 24 h.

### Treatment protocol

On admission, blood samples were drawn to estimate complete blood cell count, serum troponin I, creatinine and lipid profile and a urine sample was collected for estimation of urine-NGAL. Immediately after PPCI, patients were transferred from the catheterization laboratory to the medical ICU for continuous ECG-monitoring and pulse oximetry. On admission to the ICU, a clinical examination and standard ECG were performed and the patency of peripheral intravenous catheter, inserted before the PPCI, was checked. In case of hemodynamic compromise, the treating physician assessed the need for invasive monitoring. Combined antiplatelet therapy (acetylsalicylic acid and clopidogrel), initiated either in the emergency department or by outpatient emergency units, was continued, statins and angiotensin-converting enzyme inhibitors were administered to all patients unless contraindicated. After admission to the ICU, all patients were started on intravenous saline infusions 0.5 to 1.0 ml/kg/hour, the total amount of fluid administered depended on clinical signs of acute pulmonary congestion [14].

Complete blood cell count, troponin I, serum creatinine, N-terminal pro brain natriuretic peptide (NT-proBNP) and CRP levels were estimated daily from blood samples collected between 8.00 and 10.00 a.m. in the first two days, later these tests were performed at the discretion of the treating physician. For the study purposes, a urine sample was taken for estimation of NGAL 12 h after admission and serum creatinine level was estimated every 6 h during the first 24 h. Two dimensional transthoracic echocardiography was performed within the first 24–48 h. The biplane method of disks (modified Simpson’s rule) was used to calculate left ventricular ejection fraction (EF); values higher than 55 % are considered normal [15]. Other investigations and treatment modalities were indicated by the ICU team individually for each patient. During treatment, we obtained data on prior known arterial hypertension, diabetes, dyslipidemia, prior stroke, prior MI, smoking and physical activity.

For the purpose of this study, HF was defined as NT-proBNP ≥ 400 pmol/l, in accordance with the European guidelines [16]. Other estimates of heart failure were the Kilip-Kimbal classification and ejection fraction of the left ventricle.

### Laboratory methods

The lipid profile - total serum cholesterol, HDL-cholesterol and triglycerides were estimated by the colorimetric method (Ektachem 250 Analyzer, Eastman Kodak Company, Rochester, USA). Urine NGAL by the chemiluminiscent microparticle immunoassay (CMIA) for the quantitative determination of neutrophil gelatinase-associated lipocalin in human urine (Abbott ARCHITECTAnalyzer, Abbott Ireland Diagnostics Division, Lisnamuck, Ireland; normal levels up to 131.7 ng/ml – coefficient of variability 4.6 %). Troponin I was estimated by colorimetric-immune method (Siemens Healthcare Diagnostics Inc., Newark, USA, normal levels up to 0.045 μg/l). Plasma NT-proBNP levels was estimated by the electrochemiluminescence immunoassay on an Elecsys 2010 analyzer (Roche Diagnostics, normal levels up to 20 pmol/L). Complete blood cell count was performed by automatic counter Sysmex XE - 2100, Kobe Japan; normal levels for leukocytes were 4 – 10 × 10^9^/l, erythrocytes 4.2 – 6.3 × 10^12^/l and platelets 140 – 340 × 10^9^/l.

### Statistical analysis

Demographic characteristic and laboratory data were analysed using descriptive methods – frequencies, mean with standard deviation or median with interquartile range. Subgroup comparisons were done using *χ*^2^ test for categorical and Student’s *t*-test or the nonparametric Mann–Whitney test for numeric variables. Bivariate correlations were tested by Spearmann’s coefficient (rho). P value of 0.05 was set as the limit of statistical significance. SPSS Statistics 18.0 (IBM ®) was used for statistical analysis.

Sensitivity and specificity of NGAL concentration for heart failure defined by elevated NT-proBNP level was calculated [16]. A receiver operating characteristic (ROC) diagram was constructed with urine NGAL level as the test variable and NT-proBNP level as the state variable (Fig. 3). Area under the curve of the constructed ROC diagram was 0.67 (95 % CI 0.52, 0.83; *p* = 0.02). Although the area under the curve was relatively low, it still was considered relevant. The cut-off of urine NGAL with 90 % specificity for heart failure was 46.95 ng/ml, the sensitivity of this cut-off was 25 %; the value 50 ng/ml was used in addition to the previously established normal value of 137 ng/ml. This 50 ng/ml cut-off was thus derived from the same dataset as used in all other statistical analyses.

Urine NGAL levels, markers of kidney injury and markers of inflammation were compared between predefined subgroups of patients with NT-proBNP < 400 pmol/l and patients with NT-proBNP ≥ 400 pmol/l. Conversely, markers of heart failure, inflammation and kidney injury were compared in subgroups based on values of urine NGAL. A linear regression model was constructed with NT-proBNP as the dependent and age, gender, NGAL level, CRP, peak creatinine and left ventricular ejection fraction as independent variables.

## Results

**Fig. 1:**
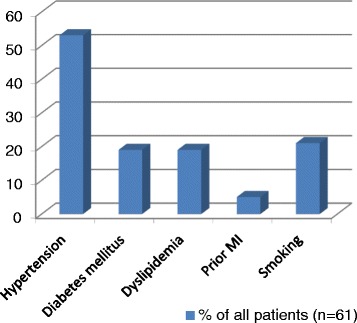
Risk factors for vascular diseases in study participants. Legend: MI = myocardial infarction

In total, 61 patients were included in the study, among them 44 (72 %) men. The mean age was 63.8 years (SD 12.8 years) As shown in Fig. 1, the most prevalent risk factor for vascular disease was arterial hypertension, followed by dyslipidemia and diabetes.

**Fig. 2:**
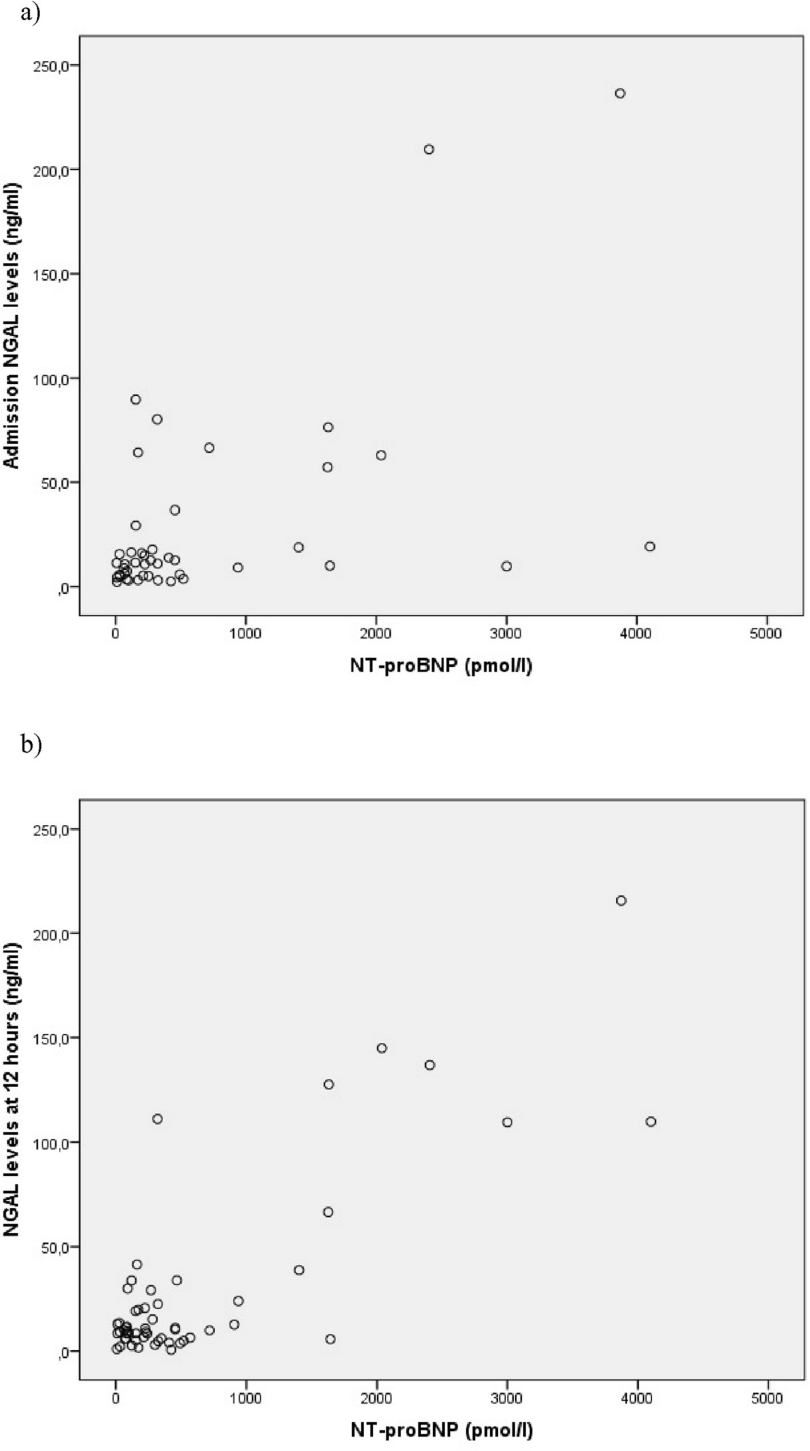
The distribution of urine neutrophil gelatinase-associated lipocalin (NGAL) concentration at admission (**a**) and 12 h after admission (**b**) and its relation to serum N-terminal pro brain natriuretic peptide (NT-proBNP) concentration. **a** Urine NGAL levels at admission. **b** Urine NGAL levels 12 h after admission. Legend: NGAL = neutrophil gelatinase-associated lipocalin; NT – proBNP = N-terminal pro brain natriuretic peptide

Among the studied patients, 39 were in Kilip class I, 11 in Kilip class II, 5 in Kilip class III and 6 in Kilip class IV as the higher class during their stay in the ICU. The average values of analysed laboratory variables in the whole sample are presented in Table 1 and the distribution of NGAL levels stratified by heart failure is presented in Fig. 2. There was no significant difference between NGAL levels at admission and after 12 h and, overall, the levels were well below the 137 ng/ml as the usual normal cut-off value. Only two patients had urine NGAL above 137 ng/ml both at admission and after 12 h.

**Table 1:** Admission and in-hospital laboratory data of the included STEMI patients

Laboratory data (mean ± SD)	*N* = *61*
Urine NGAL at admission (ng/ml)	24.5 ± 42.1
Urine NGAL at 12 h (ng/ml)	27.7 ± 42.7
In-hospital NT-proBNP (pmol/l)	565.0 ± 882.2
EF (%)	42.2 ± 12.5
Troponin I at admission (μg/l)	12.1 ± 20.5
Troponin I after 12 h (μg/l)	55.7 ± 37.0
CRP at 24 h (mg/l)	99.0 ± 54.7
Creatinine at admission (μmol/l)	102.5 ± 45.0
Creatinine at 24 h (μmol/l)	99.0 ± 54.7
Leucocyte count at 24 h (1 × 10^12^/l)	10.9 ± 3.9
Contrast material infused (ml)	147.2 ± 67.6

Data are presented as mean ± standard deviation

*MI* myocardial infarction, *NGAL* neutrophil gelatinase-associated lipocalin, *CRP* C reactive protein, *EF* left ventricular ejection fraction, *NT* – *proBNP* N-terminal pro brain natriuretic peptide

We compared markers of inflammation and of cardiac function in patients with NT-proBNP levels above and below 400 pmol/l which was the definition for heart failure in or study ([16]; Table 2). There was a significant difference between subgroups in urine NGAL levels and in markers of inflammation. The subgroups did not differ in the amount of radiologic contrast material infused.

**Table 2:** Comparison of subgroups with heart failure and with no heart failure as defined by N-terminal pro brain natriuretic peptide level (NT-proBNP)

Variables (mean ± SD)	NT-proBNP < 400 pmol/l (*n* = 41)	NT-proBNP ≥ 400 pmol/l (*n* = 20)	P value
EF at 24–48 h (%)	48.1 ± 7.8	31.3 ± 11.9	<0.01
Urine NGAL at admission (ng/ml)	14.8 ± 19.2	45.0 ± 65.2	<0.01
Urine NGAL at 12 h (ng/ml)	15.5 ± 18.3	53.8 ± 63.6	<0.01
Troponin I at admission (μg/l)	7.2 ± 13.0	22.5 ± 28.7	<0.01
Creatinine at 24 h (μmol/l)	83.7 ± 28.9	134.0 ± 79.9	<0.01
CRP at 24 h (mg/l)	21.3 ± 23.3	59.5 ± 74.9	<0.01
Leucocytes at 24 h (1 × 10^12^/l)	10.1 ± 3.0	12.6 ± 5.1	<0.01
Amount of contrast material (ml)	142.0 ± 67.3	160.3 ± 69.6	NS

Data are presented as mean ± standard deviation

*MI* myocardial infarction, *NGAL* neutrophil gelatinase-associated lipocalin, *CRP* C reactive protein, *EF* left ventricular ejection fraction, *NT*-*proBNP* N-terminal pro brain natriuretic peptide

**Fig. 3:**
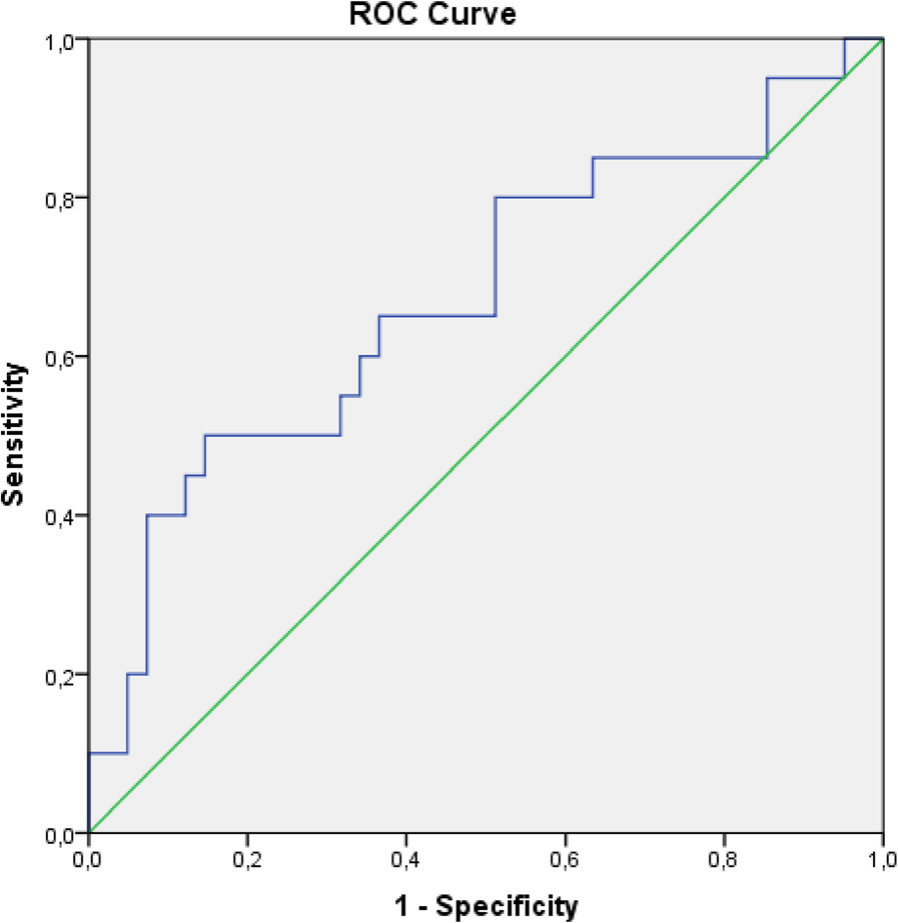
The receiver operating characteristic (ROC) curve for determination of cut-off value and predictive values of neutrophil gelatinase-associated lipocalin (NGAL) in urine for heart failure in patients after myocardial infarction

As explained in the methods section, urine NGAL 50 ng/ml was established as the level with 90 % specificity for acute heart failure defined by NT-proBNP (Fig. 3). Comparison of the two subgroups with NGAL levels < 50 ng/ml and ≥ 50 ng/ml at admission and after 12 h revealed a significant difference in NT-proBNP levels and in ejection fraction as well as in markers of inflammation and of kidney function between subgroups (Table 3). When we used 137 ng/ml as the cut-off value, subgroups did not differ in any of the analyzed variables.

**Table 3:** Admission and in-hospital laboratory findings by urine NGAL

Variables	Admission urine NGAL < 50 ng/ml (*n* = 52)	Admission urine NGAL ≥50 ng/ml (*n* = 9)	*p*	Urine NGAL after 12 h < 50 ng/ml (*n* = 52)	Urine NGAL after 12 h ≥ 50 ng/ml (*n* = 9)	*p*
NT-proBNP after 24 h (pmol/l)	218.0 (82.5–420.0)	1627.0 (245.50–2221.5)	*p* < 0.05	214.0 (84.0–424.0)	2221.5 (1628.0–3654.0)	*p* < 0.05
EF (%)	47 (35–50)	25 (20–40)	*p* < 0.05	45 (35–50)	20 (20–36.2)	*p* < 0.05
Kilip ≥ 2 (%)	22	78	*p* < 0.05	22	78	*p* < 0.05
Leucocytes after 24 h (1 × 1012/l)	9.2 (7.8–12.1)	14.3 (8.5–16.3)	NS	9.2 (7.8–11.6)	14.3 (8.9–16.5)	NS
CRP after 24 h (mg/l)	16.0 (10.0–30.0)	32.0 (6.5–140.5)	NS	15.0 (7–28.5)	80.0 (36.0–177.8)	*p* < 0.05
Creatinine admission (μmol/l)	91.0 (69.8–114.0)	140.0 (100.0–216.0)	*p* < 0.05	92.0 (74.0–114.0)	153.0 (111.0–217.0)	*p* < 0.05
Creatinine after 24 h (μmol/l)	78.5 (63.0–94.5)	142.0 (94.5–273.0)	*p* < 0.05	78.0 (63.5–93.5)	198.5 (133.8–275)	*p* < 0.05
Contrast material (ml)	140.0 (110.0–178.6)	90.0 (61.5–198.8)	NS	135.0 (100.0–168.0)	174.2 (110.0–240.0)	NS

Data are presented as median (interquartile range) for numeric and as proportions for categorical variables

*NGAL* neutrophil gelatinase-associated lipocalin, *CRP* C reactive protein, *EF* left ventricular ejection fraction, *NT* – *proBNP* N-terminal pro brain natriuretic peptide

Because NGAL has high specificity for heart failure after STEMI in our sample, we attempted to establish correlations between NGAL levels and markers of inflammation and of heart failure. There was a positive association between admission urine NGAL and in-hospital NT-proBNP (rho_s_ 0.34, *p* = 0.003) and between admission urine NGAL and in-hospital CRP (rho_s_ 0.22, *p* = 0.04). Results were similar for urine NGAL estimated 12 h after admission (rho_s_ 0.34 for NT-proBNP, *p* = 0.003 and rho_s_ 0.353 for CRP, *p* = 0.03). There was a negative association between NGAL on admission and EF (rhos – 0.122, *p* = 0.19). In a multiple linear regression model, urine NGAL levels (β = 8.91) and female gender (β = 464.1) were independently associated with NT-proBNP concentration, while age, peak troponin, creatinine and CRP were not; adjusted r^2^ for model 0.716.

## Discussion

In our sample of patients with acute STEMI we observed NGAL levels below the usual normal limit 137 ng/ml in the great majority of participants. However, higher levels of NGAL – even though below the above stated limit – had high specificity for development of heart failure and urine NGAL concentration was positively correlated with serum concentration of NT-proBNP. These results suggest that elevated urine NGAL very early after STEMI could be a marker of high risk for acute heart failure in an individual patient and that with regard to MI much lower concentrations of NGAL are of significance compared to when evaluating patients with kidney failure [17].

Urine NGAL was individually correlated with NT-proBNP as well as with CRP and creatinine. In a multiple linear regression model, NGAL was an independent predictor of NT-proBNP concentration, even though in clinical terms the association was weak. This association and the correlation between NGAL and CRP can be viewed as evidence for the importance of early inflammation in acute heart failure after MI. But we must state that NGAL has several possible sources – most commonly is has been associated with inflammation and with kidney dysfunction – and that our study design does not allow deductions about causality.

Damman et al. have shown that increased urine NGAL is highly prevalent in patients with chronic HF and, similarly to our results, positively associated with increased levels of NT-proBNP [18]. Other authors have demonstrated that in patients with chronic HF of ischemic origin with normal renal function, serum NGAL levels were markedly increased in comparison to controls and this increase independently correlated with New York heart Association (NYHA) class and the estimated glomerular filtration rate [19]. It has also been demonstrated that plasma NGAL strongly predicted mortality in patients with chronic heart failure with and without chronic kidney disease [20]. Our results suggest that NGAL is also associated with markers of acute HF also in the setting of STEMI and this in probably independent of kidney function. Previous (chronic) heart failure is a possible confounding factor, but unfortunately, we did not have reliable enough data about past cardiac function in our participants.

Kidney damage is another possible cause of elevated NGAL. After STEMI, kidney injury may be the consequence of different mechanisms including systolic dysfunction, nephrotoxicity of contrast material used during PPCI, other nephrotoxic drugs, activation of sympathetic and renin-angiotensin-aldosterone system, inflammation and oxidative stress. NGAL can be released by activated neutrophil, epithelial and endothelial cells, smooth muscle cells and cells in in atherosclerotic plaques [19, 21]. Aghel et al. revealed that patients who developed worsening renal function during hospitalization had significantly higher median admission serum NGAL levels (admission NGAL levels ≥ 140 ng/ml) [22]. In our patients, the mean creatinine level was near normal and no one required renal replacement therapy. The amount of contrast material did not differ between patients with and without heart failure nor between patients with NGAL levels above and below 50 ng/ml and we therefore assume that kidney failure is unlikely the sole explanation for elevated NGAL in patients with heart failure in our sample. Palazzuoli et al. showed that NGAL levels above 130 ng/ml predicted not only renal injury, but also an overall risk for cardiac events [17]. According to our data, the NGAL levels predicting heart failure are even lower.

### Study limitations

Several limitations may influence our results. We studied urine NGAL concentrations, which may follow a different timeline than serum concentration and have a different relationship with kidney damage compared to serum concentration. We also had no data on prior heart failure, which has been shown to influence NGAL levels [18]. Importantly, the cut-off value of urine NGAL with high specificity for HF (50 ng/ml) was derived from the analyzed sample itself and this will have to be verified in other, larger samples. Patients who were discharged from the ICU within 24 h were excluded from the study and this diminished the number of participants with a possible bias towards more severe cases.

## Conclusions

The level of urine NGAL early after myocardial infarction is associated with NT-proBNP concentration and even NGAL levels below 137 ng/ml, the usually reported normal cut-off value, had high specificity for HF in our sample. The mechanism of urine NGAL increase after STEMI and its potential value as a predicting factor for acute HF should be investigated in further studies.
